# A Ressonância Magnética e Tomografia Computadorizada Cardiovascular na Cardiologia do Presente e Futuro

**DOI:** 10.36660/abc.20230021

**Published:** 2023-02-17

**Authors:** Carlos E. Rochitte

**Affiliations:** 1 Hospital das Clínicas Faculdade de Medicina Universidade de São Paulo São Paulo SP Brasil Instituto do Coração do Hospital das Clínicas da Faculdade de Medicina da Universidade de São Paulo – (InCor, HCFMUSP), São Paulo, SP – Brasil; 2 Hospital do Coração São Paulo SP Brasil Hospital do Coração (HCOR), São Paulo, SP – Brasil

**Keywords:** Diagnóstico por Imagem/tendências/métodos, Doença Arterial Coronariana, Aterosclerose, Estenose Coronária, Diagnóstico Precoce, Fenótipo, Genótipo

Métodos de imagem não invasivos modernos, como a tomografia computadorizada cardiovascular (TCC) e a ressonância magnética cardiovascular (RMC), têm a capacidade de diagnosticar e monitorizar uma ampla gama de doenças cardiovasculares com acurácia, precisão e segurança sem precedentes.^1 - 3^ Com a evolução do entendimento sobre as doenças cardiovasculares e a disponibilidade de novos e revolucionários tratamentos, informações detalhadas e quantitativas sobre o estágio da doença passaram a ser fundamentais para a tomada de decisão mais apropriada para cada paciente, um dos pilares da medicina personalizada.

**Figura 1:**
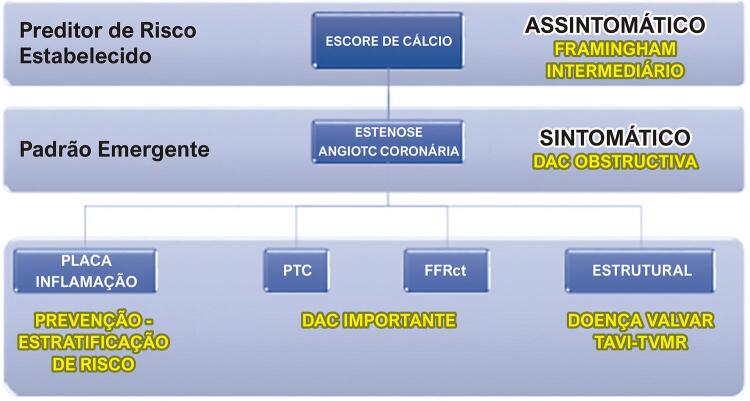
– Indicações da tomografia computadorizada cardiovascular. AngioTC: angiotomografia; DAC: doença arterial coronária; PTC: perfusão miocárdica por tomografia computadorizada; FFRct: reserva de fluxo fracional por tomografia computadorizada; TAVI: implante transcateter de válvula aórtica.

Como exemplo, podemos citar a alta acurácia da TCC no diagnóstico e na quantificação da aterosclerose e estenose coronárias ( Figura 1 ), e a avaliação da significância funcional pela técnica de reserva de fluxo fracional ( *fractional flow reserve* , FFR) por TC. Considerando os novos conhecimentos de que características específicas da placa e a carga global de placa da árvore coronária podem ser indicadores poderosos do prognóstico da doença arterial coronariana (DAC), a TCC passa a ser peça fundamental na decisão terapêutica, seja para a revascularização de estenoses, seja na prevenção de eventos cardiovasculares adversos. Um outro exemplo da TCC é a capacidade de monitorizar a resposta das placas ateroscleróticas a terapias avançadas como aquelas envolvendo a interferência na PCSK9 por anticorpos monoclonais ou por RNA de interferência.^1 - 3 , 4 - 6^

**Figura 2:**
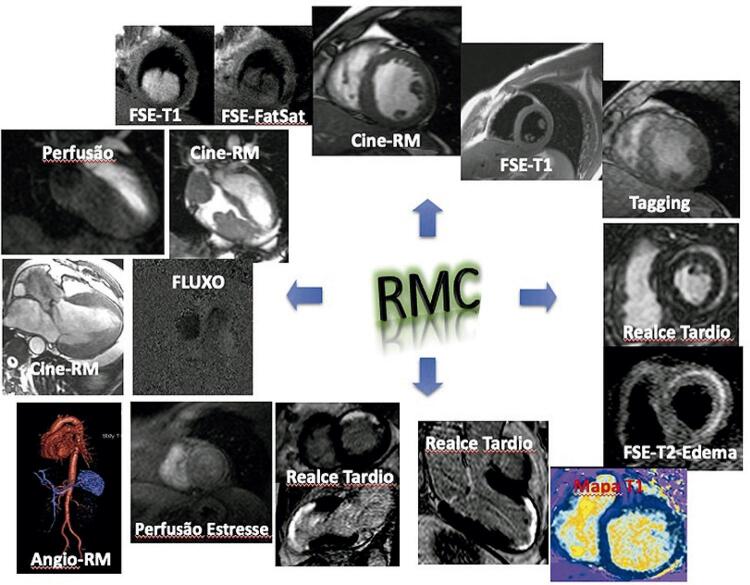
– Técnicas e indicações clínicas de Ressonância Magnética Cardiovascular. RM: ressonância magnética; FSE: Spin-eco rápido; FatSat: saturação de gordura.

Ainda na DAC, a RMC permite a investigação detalhada do ventrículo esquerdo e seu miocárdio, com técnicas que incluem a avaliação da função contrátil global e regional ( *strain* miocárdicos), visualização do infarto e quantificação da viabilidade miocárdica de forma precisa, e a perfusão miocárdica durante estresse que permite a detecção de defeitos perfusionais associados a estenoses coronárias hemodinamicamente significativas ( Figura 2 ). Mais recentemente, a RMC pode quantificar o fluxo miocárdico absoluto em mL/min/g em repouso e estresse, permitindo o cálculo da reserva de fluxo coronário ( *coronary flow reserve* , CFR), algo antes só possível por técnicas mais complexas como o PET/TC. O CFR é atualmente considerado o parâmetro mais preciso para caracterizar isquemia miocárdica, sendo essencial na definição de doença microvascular (INOCA, *ischaemia with no obstructive coronary artery disease* ), quando não há estenose coronária significativa detectável.^1 - 3 , 7^

Com estas breves explanações, fica claro que a parceria da RMC e TCC já é fundamental na Cardiologia avançada atual e serão pilares essenciais da Cardiologia do futuro, na assistência e desenvolvimento.

Além da DAC, no âmbito das cardiomiopatias (CMP) e da insuficiência cardíaca (IC), as técnicas de RMC e TCC têm sido mais utilizadas, objetivando diagnósticos mais precoces, mais precisos e quantitativos, o que leva à escolha de terapias mais apropriadas. Na IC, como síndrome clínica, a RMC e a TCC podem avaliar parâmetros quantitativos da função ventricular direita e esquerda, geometria ventricular, contratilidade regional específica ( *strain* miocárdicos), volume e função atrial, em particular do átrio esquerdo. No diagnóstico de CMP, além dos parâmetros citados para IC, a caracterização tecidual do miocárdio passa a ter papel fundamental, seja ela pelo clássico realce tardio, seja pela avaliação quantitativa da matriz intersticial do miocárdico com os cálculos do T1 (tempo de relaxamento longitudinal), T2 (relaxamento transversal) e volume extracelular do miocárdio ( *extracellular volume* , ECV). Os mapas paramétricos do T1 pré e pós-contraste fornecem o cálculo do ECV e, associados ao T2 miocárdico, podem dar uma visão abrangente da microestrutura do miocárdio em suas alterações mesmo que discretas. Importante destacar que embora esses parâmetros sejam avaliados classicamente pela RMC, a TCC também pode avaliar o realce tardio miocárdico e o ECV de forma precisa.^1 - 3 , 8 - 14^

Ainda no diagnóstico das CMP, as técnicas de caracterização tecidual pela RMC e TCC são hoje peças-chaves nas fases iniciais da doença. Como exemplo, na CMP dilatada, a presença de realce tardio do tipo *ring-like* eleva em muito o risco arritmogênico maligno desta entidade. Nas CMP que cursam com hipertrofia ventricular, e nas quais os diferenciais de hipertrofia incluem cardiomiopatia hipertrófica, CMP infiltrativa ou de depósitos, a RMC tem um papel crucial no diagnóstico e estadiamento da doença. Recentemente, por exemplo, a amiloidose com envolvimento cardíaco tem sido diagnosticada de forma muito mais frequente, precoce e mesmo sem necessidade de biópsia, utilizando técnicas de multimodalidade de imagem cardiovascular. A RMC tem papel central no diagnóstico e único no seguimento e monitorização da carga amiloide durante tratamento.^10 , 12^ Na maioria das doenças cardiovasculares, pouquíssimos aspectos não são avaliados pela combinação destes dois poderosos métodos de imagem cardiovascular não invasivos, a RMC e a TCC.

Este editorial reforça o que muitos autores e sociedades médicas de cardiologia têm enfatizado, a RMC e TCC são os instrumentos fundamentais para a fenotipagem avançada das doenças cardiovasculares. O fenótipo avançado correto em associação com a síndrome clínica e o genótipo (em algumas situações específicas) são a base para a escolha do melhor tratamento personalizado das doenças cardiovasculares. A Cardiologia, em nível terciário de atenção, não pode prescindir da RMC e TCC, o que tem sido demonstrado por inúmeras diretrizes clínicas, regulamentações oficiais, e mesmo nas descrições de programas da disciplina de Cardiologia de universidades de ponta no Brasil e no exterior.^15 , 16^

O modelo clínico-fenotípico-genotípico tem sido corroborado pelas evidências científicas e avança rapidamente para uma adoção ampla na Cardiologia do futuro próximo.
