# Supplementary material for: A Ressonância Magnética e Tomografia Computadorizada Cardiovascular na Cardiologia do Presente e Futuro
Source: Arq Bras Cardiol. 2023 Feb 17;120(1):e20230021. [Article in Portuguese] doi: 10.36660/abc.20230021 (PMC12080721; doi:10.36660/abc.20230021)
Supplement: Translation

# Translation: Cardiovascular Magnetic Resonance and Cardiovascular Computed Tomography in the Present and Future Cardiology

**Authors:** Carlos E. Rochitte

**Affiliations:** 1 Brazil Instituto do Coração do Hospital das Clínicas da Faculdade de Medicina da Universidade de São Paulo – (InCor, HCFMUSP), São Paulo, SP – Brazil; 2 Brazil Hospital do Coração (HCOR), São Paulo, SP – Brazil

**Keywords:** Diagnostic Imaging/trends/methods, Coronary Artery Disease, Atherosclerosis, Coronary Stenosis, Early Diagnosis, Fhenotype, Genotype

Modern non-invasive imaging techniques like cardiovascular computed tomography (CCT) and cardiovascular magnetic resonance (CMR) can diagnose and monitor a wide range of cardiovascular diseases with unprecedented accuracy and safety.^1 - 3^ With advances in the understanding of cardiovascular diseases and increasing availability of new and revolutionary therapies, detailed and quantitative information of disease stage have become crucial for the appropriate decision-making for each patient, which is one of the pillars of personalized medicine.

As an example, we can mention the high accuracy of CCT in the diagnosis and quantification of coronary atherosclerosis and stenosis ( Figure 1 ), and assessment of functional significance by the fractional flow reserve (FFR) technique. Considering recent knowledge of the role of characteristics of the plaque and the global burden of the atherosclerotic plaque involving the coronary tree on the prognosis of coronary artery disease (CAD), CCT has turned out to be a key tool in therapeutic decision making, be it for stenosis revascularization, be it for prevention of adverse cardiovascular events. Another example is the ability of CCT in monitoring the response of atherosclerotic plaques to advanced therapies, like those including PCSK9 inhibition by monoclonal antibodies or RNA interference.^1 - 3 , 4 - 6^

**Figure 1:** – Cardiovascular computed tomography indications. CTA: coronary computed tomography angiography; CTP: computed tomography perfusion; FFRct: fractional flow reserve by computed tomography; TAVI: transcatheter aortic valve implant/replacement; TVMR: transcatheter mitral valve replacement; CAD: coronary artery disease.

Also in CAD, CMR allows a detailed examination of the left ventricle and its myocardium, by techniques that include the assessment of global and regional contraction (myocardial strain), accurate visualization of infarction and quantification of myocardial viability, and myocardial perfusion during stress, which allows the detection of perfusion defects associated with hemodynamically significant coronary stenosis ( Figure 2 ). More recently, CMR has been used to quantify absolute myocardial flow (mL/min/g), both at rest and during stress, enabling the calculation of coronary flow reserve (CFR), which was only possible via more complex techniques, like PET/CT. CFR is currently considered the most accurate parameter for characterizing myocardial ischemia, being fundamental in defining coronary microvascular disease (INOCA, ischemia with no obstructive coronary artery disease), when there is no detectable significant coronary stenosis.^1 - 3 , 7^

**Figure 2:** – Cardiovascular Magnetic Resonance techniques and indications. LGE: late gadolinium enhancement; MRA: magnetic resonance angiography; MR: magnetic resonance; FSE: fast spin-echo; FatSat: fat saturation.

Based on the above, the partnership between CMR and CCT is already crucial in current advanced cardiology and will be essential pillars of future cardiology, in terms of care and development.

In addition to CAD, CMR and CCT have been increasingly used in cardiomyopathies (CMP) and heart failure (HF), focusing on earlier and more precise quantitative diagnosis, that lead to more appropriate therapeutic choices. In HF, as a clinical syndrome, both CMR and CCT can examine quantitative parameters of right and left ventricular function, ventricular geometry, regional contractility (myocardial strain), atrial volume and function, particularly in the left atrium. In the diagnosis of CMP, in addition to the parameters for HF, myocardial tissue characterization plays a crucial role, for the classical myocardial late enhancement and for the quantitative assessment of myocardial interstitial matrix by calculation of T1 (longitudinal relaxation time), T2 (transversal relaxation time), and myocardial extracellular volume (ECV). Pre- and post-contrast parametric maps at T1 enable the ECV estimation and, combined with myocardial T2, can provide a comprehensive view of myocardial microstructure and its changes, even discrete ones. It is worth pointing out that, although these parameters are classically evaluated by CMR, CCT can also precisely evaluate myocardial late enhancement and ECV.^1 - 3 , 8 - 14^

Also in the context of CMP, the techniques of tissue characterization by CMR and CCT are key elements in the initial stages of the disease. As an example, in dilated CMP, the presence of a ring-like late gadolinium enhancement greatly increases the risk of malignant arrhythmia. In CMP that progress to ventricular hypertrophy, and that differential diagnoses of hypertrophy include hypertrophic, infiltrative or deposition CMP, CMR plays a crucial role in disease diagnosis and staging. For example, the diagnosis of amyloidosis with cardiac involvement has been made more frequently and earlier, even without the need of biopsy, using multimodal cardiovascular imaging techniques. CMR plays a central and unique role in the follow-up and monitoring of amyloid burden during treatment.^10 , 12^ In most cardiovascular diseases, very few aspects cannot be evaluated by the combination of these two powerful non-invasive cardiovascular imaging techniques – CMR and CCT.

This editorial reinforces what many authors and cardiology societies have highlighted – CMR and CCT are fundamental tools for the advanced phenotyping of cardiovascular diseases. A correct advanced phenotyping, in combination with the clinical syndrome and genotyping (in some cases) form the basis of choosing the best individualized treatment for cardiovascular disease patients. Both CMR and CCT are indispensable in settings of tertiary cardiology care; this has been emphasized in several guidelines, official regulations, and even in cardiology disciplines offered in top-notch universities in Brazil and other countries.^15 , 16^

The clinical-phenotype-genotype model has been supported by scientific evidence and has rapidly advanced towards a wide-scale use in near future.
